# The glycine receptor alpha 3 subunit mRNA expression shows sex-dependent differences in the adult mouse brain

**DOI:** 10.1186/s12868-023-00800-9

**Published:** 2023-06-01

**Authors:** Mikaela M. Ceder, Hannah M. Weman, Ebba Johansson, Katharina Henriksson, Kajsa A. Magnusson, Erika Roman, Malin C. Lagerström

**Affiliations:** 1 Department of Immunology, Genetics and Pathology, Uppsala University, Uppsala, Sweden; 2 Department of Anatomy, Physiology and Biochemistry, Swedish University of Agricultural Sciences, Uppsala, Sweden; 3 Neuropharmacology and Addiction, Department of Pharmaceutical Biosciences, Uppsala University, Uppsala, Sweden

**Keywords:** Glycine, Glra3, Brain, Spinal cord, Mice, Sex-dependent differences

## Abstract

**Background:**

The glycinergic system plays an important inhibitory role in the mouse central nervous system, where glycine controls the excitability of spinal itch- and pain-mediating neurons. Impairments of the glycine receptors can cause motor and sensory deficits. Glycine exerts inhibition through interaction with ligand-gated ion channels composed of alpha and beta subunits. We have investigated the mRNA expression of the glycine receptor alpha 3 (*Glra3*) subunit in the nervous system as well as in several peripheral organs of female and male mice.

**Results:**

Single-cell RNA sequencing (scRNA-seq) data analysis on the Zeisel et al. (2018) dataset indicated widespread but low expression of *Glra3* in vesicular glutamate transporter 2 (*Vglut2, Slc17a6*) positive and vesicular inhibitory amino acid transporter (*Viaat, Slc32a1*)positive neurons of the mouse central nervous system. Highest occurrence of *Glra3* expression was identified in the cortex, amygdala, and striatal regions, as well as in the hypothalamus, brainstem and spinal cord. Bulk quantitative real-time-PCR (qRT-PCR) analysis demonstrated *Glra3* expression in cortex, amygdala, striatum, hypothalamus, thalamus, pituitary gland, hippocampus, cerebellum, brainstem, and spinal cord. Additionally, male mice expressed higher levels of *Glra3* in all investigated brain areas compared with female mice. Lastly, RNAscope spatially validated *Glra3* expression in the areas indicated by the single-cell and bulk analyses. Moreover, RNAscope analysis confirmed co-localization of *Glra3* with *Slc17a6* or *Slc32a1* in the central nervous system areas suggested from the single-cell data.

**Conclusions:**

*Glra3* expression is low but widespread in the mouse central nervous system. Clear sex-dependent differences have been identified, indicating higher levels of *Glra3* in several telencephalic and diencephalic areas, as well as in cerebellum and brainstem, in male mice compared with female mice.

**Supplementary Information:**

The online version contains supplementary material available at 10.1186/s12868-023-00800-9.

## Introduction

The amino acid glycine acts as an inhibitory neurotransmitter in mammals and contributes to the regulation of both itch- and pain-associated networks [1, 2]. Glycine is an agonist to the glycine receptors (GlyRs), which are pentameric ligand-gated ion channels that predominantly consist of four ligand binding alpha (α) subunits (GLRA1–GLRA4) and one structural beta (β) subunit [3].

The *Glra3* gene was first cloned from a rat brain cDNA library by homology screening, and found to be expressed in the spinal cord [4]. Rat *Glra3* mRNA has also been located in sub-regions of the olfactory bulb, cerebral cortex, thalamus, hippocampus, cerebellum [5], and the retina [6]. Analysis in mice has mapped the *Glra3* gene to chromosome 8 [7], and transcriptional analyses of the spinal cord and dorsal root ganglia (DRG) have located *Glra3* expression to the spinal dorsal horn. In contrast, expression of *Glra3* was not detected in DRG and below detection level in the ventral spinal horn [8]. Additionally, the Allen Institute has mapped *Glra3* to both the dorsal and ventral horn as well as to different divisions of the spinal cord (https://mousespinal.brain-map.org/imageseries/detail/100029493.html) [9]. Immunohistochemical analysis of GLRA3 expression in the mouse spinal cord has located the subunit’s expression to the dorsal horn [10, 11]. Functionally, spinal GLRA3 has a role in certain inflammatory pain states, where ablation or mutations of *Glra3* results in faster hypersensitivity recovery [10, 12, 13].

Although GLRA3 is mainly known for its role in spinal circuits, the subunit is also expressed in the developing and adult brain [14], and a connection between GLRA3, ethanol-mediated effects [15, 16] as well as respiratory rhythmic activity [17] has been reported. Immunohistochemical and electrophysiological analyses in mice have localized GLRA3 to post-synaptic sites in the inner plexiform layer of the retina [18], the nucleus accumbens [15, 19], dorsal striatum and medial prefrontal cortex pyramidal neurons of layer II/III [19], as well as to both presynaptic glycine transporter 2 (GLYT2)-expressing neurons and postsynaptic neurons in brainstem areas important for respiratory rhythms [17]. In situ hybridization also locates *Glra3* to the cortex, hypothalamus and midbrain (https://mouse.brain-map.org/experiment/show/70723453) [20]. Moreover, the expression of *Glra3* and immunohistochemical detection of GLRA3 are increased in the insular cortex of female mice in an endometriosis model [21].

The cited studies suggest that GLRA3 is expressed in several areas in the central nervous system. However, a detailed analysis of the *Glra3* mRNA expression, using complementary methods, in both sexes of mice is lacking. We have therefore investigated the expression of *Glra3* in adult female and male mice using quantitative real-time-PCR (qRT-PCR) and a sensitive fluorescent in situ hybridization method called RNAscope. Furthermore, we compared our findings with a publicly available single-cell RNA sequencing (scRNA-seq) dataset.

## Results

### *Glra3* exhibited low expression in excitatory and inhibitory neurons in several areas of the central nervous system

**Fig. 1:**
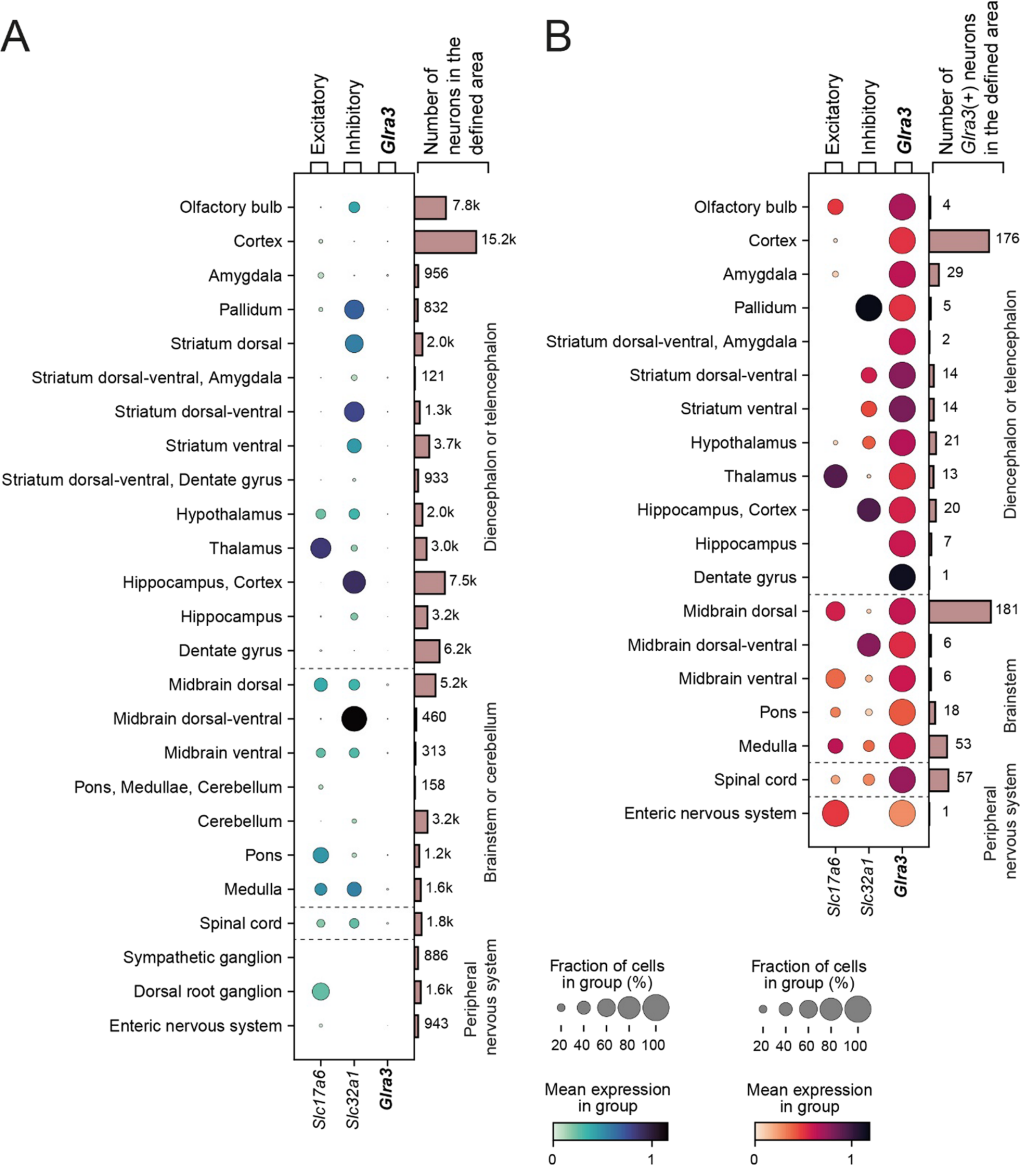
Low expression of *Glra3* was detected in the central nervous system. The expression of *Glra3* and its co-expression with excitatory marker *Slc17a6* (*Vglut2*) and inhibitory marker *Slc32a1* (*Viaat*) were examined in distinct areas in the central and peripheral nervous system in the Zeisel et al. (2018) dataset, which contained scRNA-seq data of 27,998 genes in 74,539 neurons [22]. **A** Dot plot of the expressions of the targeted genes in all neurons in the areas annotated in the Zeisel et al. (2018) dataset. *Glra3* was generally expressed in low levels, as indicated by light blue colored dots, and in a small number of cells, as indicated by the dots’ small diameters. The highest expression was found in Zeisel et al. (2018) defined central nervous system areas, namely the amygdala, dorsal midbrain, medulla and the spinal cord (more than 3.0% of neurons in these areas expressed *Glra3*). **B** The occurrence of *Glra3* neurons (*Glra3* was considered expressed if log1p > 0.1) and the co-expression of *Slc17a6* and *Slc32a1* in the respective areas visualized with a dot plot. *Glra3* was expressed in both excitatory and inhibitory neurons, with some areas displaying either excitatory or inhibitory *Glra3* neurons, while other areas contained *Glra3* neurons of both molecular properties.

Expression of *Glra3* mRNA can be detected in several areas of the brain and spinal cord [4, 5, 8]. To further investigate the expression of *Glra3* in the central and peripheral nervous system, its expression alongside expression of the excitatory marker *Slc17a6 (Vglut2)* and the inhibitory marker *Slc32a1 (Viaat)* were examined in neuronal cells from distinct nervous system areas in the Zeisel et al. (2018) scRNA-seq dataset [22]. *Glra3* in the central and peripheral nervous system showed low levels of expression (as indicated by the light-blue colored dots) and was found to be aberrantly expressed throughout cells of a given group (as indicated by the size of the dots’ diameter) (Fig. 1A, Table 1). In the brain, the highest occurrence (> 1.0%) of *Glra3* expression was found in the amygdala (3.0% expressed *Glra3*) (Fig. 1A, Table 1). Said brain region showed a small overlap between *Glra3* and *Slc17a6* expression, but no expression of *Slc32a1* was detected (Fig. 1B). Other telencephalic areas with more than 1.0% of neurons expressing *Glra3* were the cortex and some striatal regions. Expression of *Glra3* was less than 0.5% in the olfactory bulb, striatum ventral, hippocampus, and dentate gyrus (Fig. 1A, Table 1). In the cortex, the *Glra3*-expressing cells displayed low co-localization with *Slc17a6* and no overlap with *Slc32a1*. In contrast, in the striatum, both *Glra3* and *Slc32a1* were expressed, but no expression of *Slc17a6* was observed (Fig. 1B). In the diencephalon, *Glra3* expression was detected in the hypothalamus (1.1% of the neurons expressed the targeted gene in this area), where both inhibitory and excitatory neurons displayed some levels of expression (Fig. 1B). Expression of *Glra3* (0.4%) was also found in thalamic neurons, but mainly in excitatory neurons (Fig. 1B). In the brainstem, *Glra3* was found in the dorsal, dorsal–ventral and ventral midbrain neurons, with dorsal–ventral midbrain *Glra3* neurons being *Slc32a1*-expressing, and dorsal and ventral midbrain *Glra3* neurons being mainly *Slc17a6*-expressing (dorsal midbrain: 3.5%, dorsal–ventral midbrain: 1.3%; ventral midbrain: 1.9% of neurons expressed *Glra3*) (Fig. 1B, Table 1). *Glra3* was detected in 1.5% of the pons neurons and 3.4% of the medulla neurons (Fig. 1A, Table 1). In both areas, *Glra3*-positive neurons showed partial overlap with *Slc17a6* and *Slc32a1* expression (Fig. 1B). Expression of *Glra3* was not detected in the cerebellum (Fig. 1A). In the spinal cord, 3.2% of neurons were *Glra3*-positive and co-expression with both *Slc17a6* and *Slc32a1* was identified. In the peripheral nervous system, *Glra3* expression was detected in one neuron of the enteric nervous system (0.1%), and not detected in the DRG, as described in previous studies [8, 10, 11], nor in sympathetic ganglion neurons (Fig. 1A, B).

**Table 1:** Expression of *Glra3* in the distinct nervous system areas defined in the Zeisel et al. (2018) single-cell RNA sequencing dataset

Nervous system area	Total number of cells	Number of *Glra3* expressing cells*	Relative abundance of *Glra3* expression in area (%)
Olfactory bulb	7763	4	0.1
Cortex	15,205	176	1.2
Amygdala	956	29	3.0
Pallidum	832	5	0.6
Striatum dorsal	1962	ND	ND
Striatum dorsal–ventral, Amygdala	121	2	1.7
Striatum dorsal–ventral	1332	14	1.1
Striatum ventral	3676	14	0.4
Striatum dorsal–ventral, Dentate gyrus	933	ND	ND
Hypothalamus	1981	21	1.1
Thalamus	3029	13	0.4
Hippocampus, Cortex	7507	20	0.3
Hippocampus	3210	7	0.2
Dentate gyrus	6177	1	0.01
Midbrain dorsal	5204	181	3.5
Midbrain dorsal–ventral	460	6	1.3
Midbrain ventral	313	6	1.9
Pons, Medullae, Cerebellum	158	ND	ND
Cerebellum	3240	ND	ND
Pons	1196	18	1.5
Medulla	1566	53	3.4
Spinal Cord	1790	57	3.2
Sympathetic ganglion	886	ND	ND
Dorsal root ganglion	1580	ND	ND
Enteric nervous system	943	1	0.1

ND not detected. *Glra3 considered expressed if log1p < 0.1

In conclusion, the scRNA-seq analysis of the Zeisel et al. (2018) dataset revealed that *Glra3* was expressed in low levels in both excitatory and inhibitory neurons in several brain areas, as well as in the spinal cord. For more detailed information about the expression pattern of *Glra3*, see Table 1.

### *Glra3* mRNA expression was mainly found in the central nervous system

**Fig. 2:**
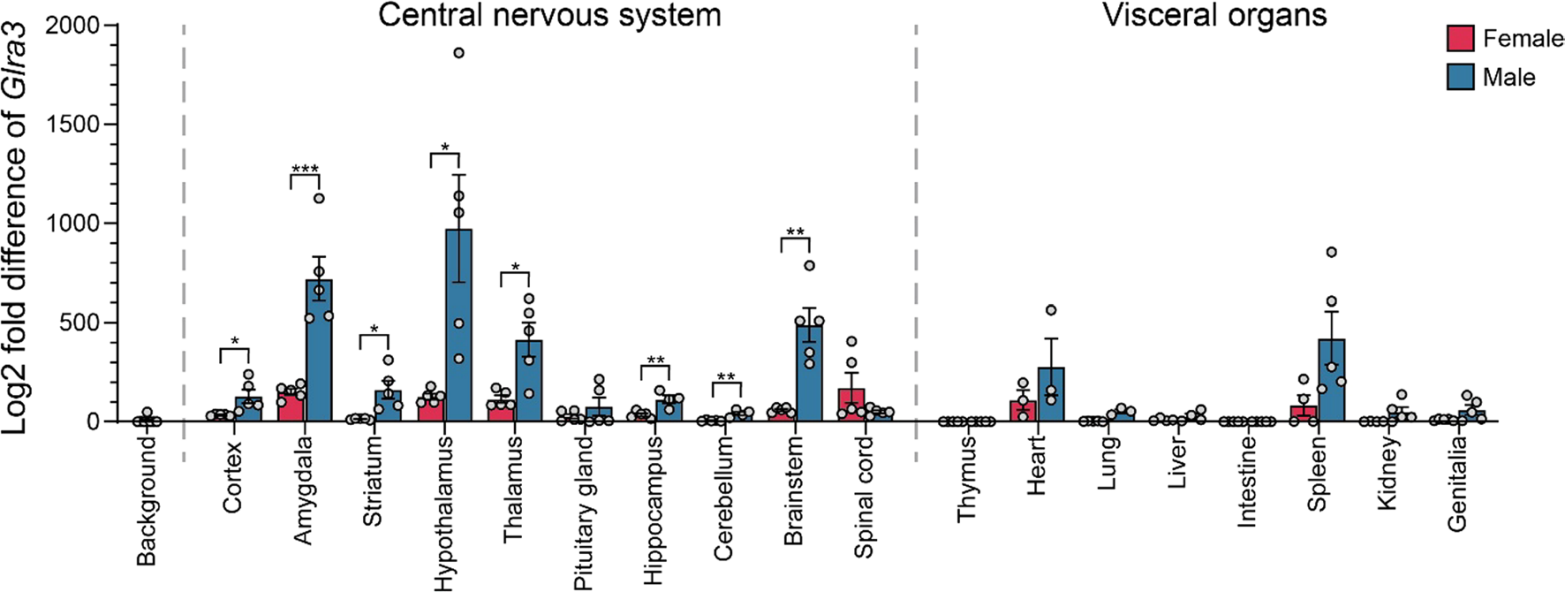
qRT-PCR revealed *Glra3* to be mainly found in the central nervous system. *Glra3* expression in adult female (n = 5, red) and adult male (n = 5, blue) C57BL/6J mice was measured using qRT-PCR, with a cutoff of 45 cycles. The relative mRNA expression was calculated using the delta Ct method with three stable reference genes (female and male body: *Actβ*, *Rpl19*, *Gapdh*; female brain: *Actβ*, *Rpl19*, *Gapdh*; male brain: *Actβ*, *Rpl19*, *Cyclo*). Stable reference genes were found using the GeNorm protocol [23]. Biological outliers (in total two outliers from male mice, one for the hippocampus and one for the cerebellum) were removed using the Grubbs outlier test with α = 0.05 before proceeding. The log2 fold mean difference (± SEM) against the genomic *Glra3* DNA expression is illustrated in the combined scatter-bar plot. *Glra3* was measured in heart (n = 3), lung (n = 3–5), spleen (n = 4–5), kidney (n = 4–5) and testes (n = 5) however these findings should be considered with caution (see Additional file 1: Fig. S1). *Glra3* was expressed in most tissues collected for the central nervous system, with highest expression in the amygdala, hypothalamus, thalamus, brainstem and spinal cord. Two-tailed Mann–Whitney U-test (thalamus p = 0.0317; hippocampus p = 0.0079 (with outlier); cerebellum p = 0.0079 (with outlier); pituitary gland p > 0.9999) or unpaired t-test (prefrontal cortex p = 0.0256; amygdala p = 0.0009; striatum (females caudate putamen, males caudate putamen and nucleus accumbens); p = 0.0118; hypothalamus p = 0.0144; hippocampus p = 0.0051 (without outlier); cerebellum p = 0.0027 (without outlier); brainstem p = 0.0010; spinal cord p = 0.1495) were used to calculate the difference between female and male mice for each tissue where *p < 0.05, **p < 0.01, ***p < 0.001

Analysis on the single-cell level revealed that *Glra3* was predominantly expressed in the cortex, amygdala, striatum, hypothalamus, brainstem and spinal cord, where *Glra3* was expressed in more than 1.0% of the neurons in each area. To broaden the analysis, we studied the relative mRNA expression of *Glra3* in both sexes of C57BL/6J mice using qRT-PCR. *Glra3* was expressed widespread in the central nervous system in both females and males(Additional file 1: Table S1). In females, highest *Glra3* expression levels were identified in the amygdala, hypothalamus, thalamus and spinal cord (top 4 areas). In comparison, male mice showed highest *Glra3* expression levels in the amygdala, hypothalamus, thalamus and brainstem (Fig. 2). Furthermore, raw cycle threshold (Ct) values (indicating expression) of *Glra3* could be detected in a few visceral organs, namely the heart and spleen, for both females and males, as well as the lung, kidney and testis tissues for male mice only. However, the measured Ct values of *Glra3* in the heart, lung, kidney, spleen and testes should be carefully reviewed, as *Glra3* was difficult to measure in the visceral organs compared with the tissues collected from the central nervous system. The relative fluorescence unit (RFU) was low, and many cycles (40–45 cycles) were needed to obtain a Ct-value, making it difficult to separate the amplification of *Glra3* in the visceral organs from background noise and non-specific amplification (Additional file 1: Fig. S1). Compared with females, males generally had higher expression of *Glra3* in the cortex (p = 0.0256), amygdala (p = 0.0009), striatum (p = 0.0118), hypothalamus (p = 0.0144), thalamus (p = 0.0317), hippocampus [p = 0.0051 (without outlier), p = 0.0079 (with outlier)], cerebellum [p = 0.0027 (without outlier), p = 0.0079 (with outlier)] and brainstem (p = 0.0010), but not in the pituitary gland (p > 0.9999) and spinal cord (p = 0.1495) (Fig. 2). In conclusion, male mice displayed higher *Glra3* mRNA levels compared with female mice in all brain areas, whereas peripheral organs expressed low to no expression of *Glra3* regardless of sex.

### *Glra3* spatial analysis corroborated with the qRT-PCR analysis

**Fig. 3:**
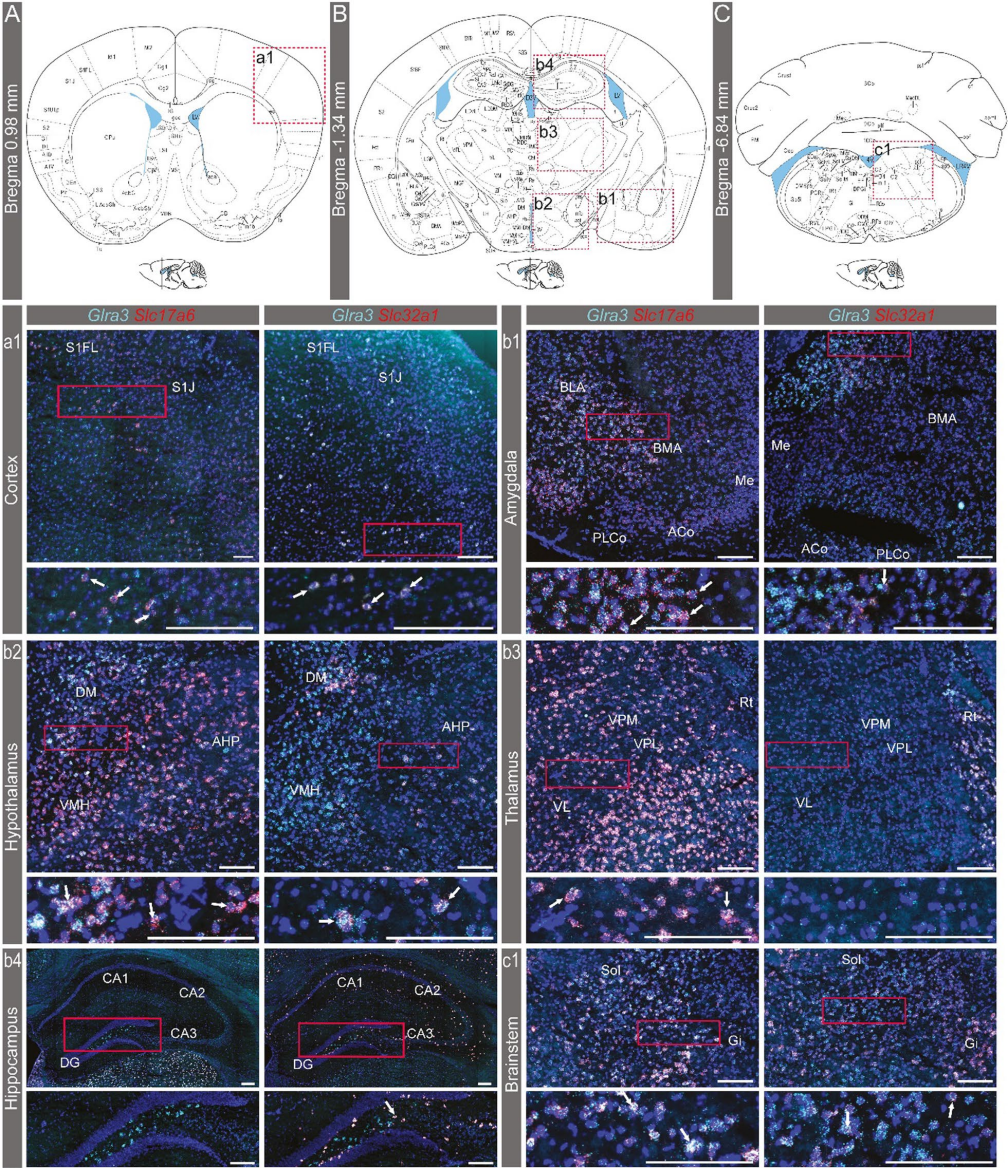
Analysis of the spatial expression of *Glra3* in the female brain. The spatial *Glra3* mRNA expression was detected with RNAscope using probes against *Glra3 (teal)*, *Slc17a6 (red)* and *Slc32a1 (red)*. Expression was detected at (**A**) Bregma 0.98 mm in the (a1) cortex, and both co-localization of *Glra3* with *Slc17a6* or *Slc32a1* was observed. **B** At Bregma -1.34 mm, *Glra3* expression was found in the (b1) amygdala, (b2) hypothalamus, (b3) thalamus and (b4) hippocampus. b1–b4 Overlap with *Slc17a6* expression was observed in all areas, except in the hippocampus, while overlap with *Slc32a1* was seen in all areas, except in the thalamus. **C** At Bregma -6.84 mm, *Glra3* expression and overlap with *Slc17a6* or *Slc32a1* expression was observed in the (c1) brainstem. Illustrations of the Bregma sections in **A–C** are adapted from https://mouse.brain-map.org/experiment/thumbnails/100048576?image_type=atlas. The red dashed squares in **A–C** indicate approximately the area displayed in a1–c1. The dashed squares were consistently placed on the right side of the schematic image in order to maximize the readability of the abbreviations, regardless of the position of the representative images. White arrows denote examples of co-expression. Scale bars: 200 µm, enlargements 100 µm. Abbreviated areas in a1–c1: Aco = anterior cortical amygdaloid nucleus, AHP = anterior hypothalamic area, posterior part, BLA = basolateral amygdaloid nucleus, anterior part, BMA = basomedial amygdaloid nucleus, anterior part, DG = dentate gyrus, DM = dorsomedial hypothalamic nucleus, Gi = gigantocellular reticular nucleus, ME = medial amygdaloid nucleus, PLCo = posterolateral cortical amygdaloid nucleus, Rt = reticular thalamic nucleus, S1FL = primary somatosensory cortex, forelimb region, S1J = primary somatosensory cortex, jaw region, Sol = solitary tract, VL = ventrolateral thalamic nucleus, VMH = ventromedial hypothalamic nucleus, VPL = ventral posterolateral thalamic nucleus, VPM = ventral posteromedial thalamic nucleus. For all other abbreviations, please see https://mouse.brain-map.org/experiment/thumbnails/100048576?image_type=atlas

**Fig. 4:**
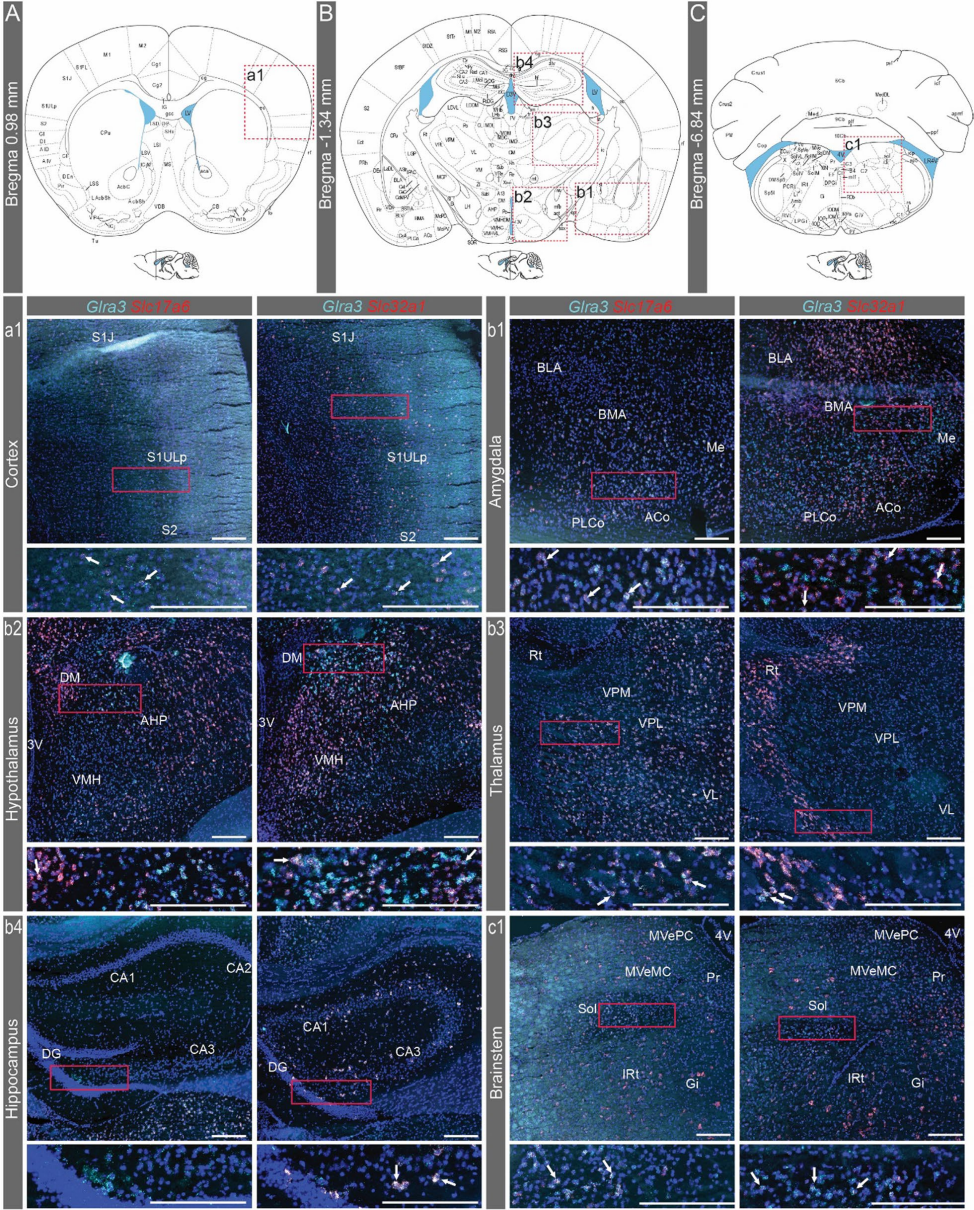
Analysis of the spatial expression of *Glra3* in the male brain. The spatial *Glra3* mRNA expression was detected with RNAscope using probes against *Glra3* (teal), *Slc17a6* (red) and *Slc32a1 (red)*. Expression was detected at (**A**) Bregma 0.98 mm in the (a1) cortex, and both co-localization of *Glra3* with *Slc17a6* or *Slc32a1* was observed. **B** At Bregma -1.34 mm, *Glra3* expression was found in the (b1) amygdala, (b2) hypothalamus, (b3) thalamus and the (b4) hippocampus (for *Slc32a1* the representative image is from a section between Bregma -1.06 and -1.22 mm). b1–b4 Overlap with *Slc17a6* expression was observed in all areas, except for the hippocampus, while overlap with *Slc32a1* expression was seen in all areas. **C** At Bregma -6.84 mm *Glra3* expression and overlap with *Slc17a6* or *Slc32a1* expressions was observed in the (c1) brainstem. Illustrations of the Bregma sections in **A–C** are adapted from https://mouse.brain-map.org/experiment/thumbnails/100048576?image_type=atlas. The red dashed squares in **A–C** indicate approximately the area displayed in a1–c1. The dashed squares were consistently placed on the right side of the schematic image in order to maximize the readability of the abbreviations, regardless of the position of the representative images. White arrows denote examples of co-expression. Scale bars: 200 µm, enlargements 100 µm. Abbreviated areas in a1–c1: Aco = anterior cortical amygdaloid nucleus, AHP = anterior hypothalamic area, posterior part, BLA = basolateral amygdaloid nucleus, anterior part, BMA = basomedial amygdaloid nucleus, anterior part, DG = dentate gyrus, DM = dorsomedial hypothalamic nucleus, Gi = gigantocellular reticular nucleus, IRt = intermediate reticular nucleus, ME = medial amygdaloid nucleus, MVeMC = medial vestibular nucleus, magnocellular part, MVePC = medial vestibular nucleus, parvicellular part, PLCo = posterolateral cortical amygdaloid nucleus, Pr = prepositus nucleus, Rt = reticular thalamic nucleus, S1J = primary somatosensory cortex, jaw region, S1ULP = primary somatosensory cortex, upper lip region, S2 = secondary somatosensory cortex, Sol = solitary tract, VL = ventrolateral thalamic nucleus, VMH = ventromedial hypothalamic nucleus, VPL = ventral posterolateral thalamic nucleus, VPM = ventral posteromedial thalamic nucleus, 3V = 3rd ventricle, 4V = 4th ventricle. For all other abbreviations, please see https://mouse.brain-map.org/experiment/thumbnails/100048576?image_type=atlas

Following *Glra3* detection in the cortex, amygdala, striatum, hypothalamus, thalamus, hippocampus, brainstem and spinal cord based on single-cell and bulk analyses, with the latter analysis revealing a sex-dependent expression pattern in several brain areas, we sought to investigate the spatial *Glra3* expression in these areas. The analysis was conducted using fluorescent in situ hybridization with the RNAscope approach [24] in female and male C57BL/6J mice, with the aim of verifying the expression of *Glra3*. The analysis was not set out to quantify *Glra3* expression and thus, no expression level comparison was performed between females and males. In both female and male brains (Figs. 3, 4), expression of *Glra3* was found in the cortex (Figs. 3A-a1, 4A-a1, somatosensory cortex displayed in the image), amygdala (Figs. 3B-b1, 4B-b1), pallidum (Additional file 1: Fig. S2A and S2a1-3), hypothalamus (Figs. 3B-b2,4B-b2), thalamus (Figs. 3B-b3, 4B-b3), hippocampus (Figs. 3B-b4, 4B-b4) and brainstem (Figs. 3C-c1, 4C-c1) areas. Expression of *Glra3* was detected in striatum of male mice, but not in females (Additional file 1: Fig. S2B). No *Glra3* probe signal could be detected in cerebellum regardless of sex (Additional file 1: Figure S2C). In all areas, except for the hippocampus, *Glra3* co-expressed with *Slc17a6*, while co-localization with *Slc32a1* was found in all brain areas except for the female thalamus (Figs. 3a1–c1, 4a1–c1, Additional file 1: Fig. S2).

**Fig. 5:**
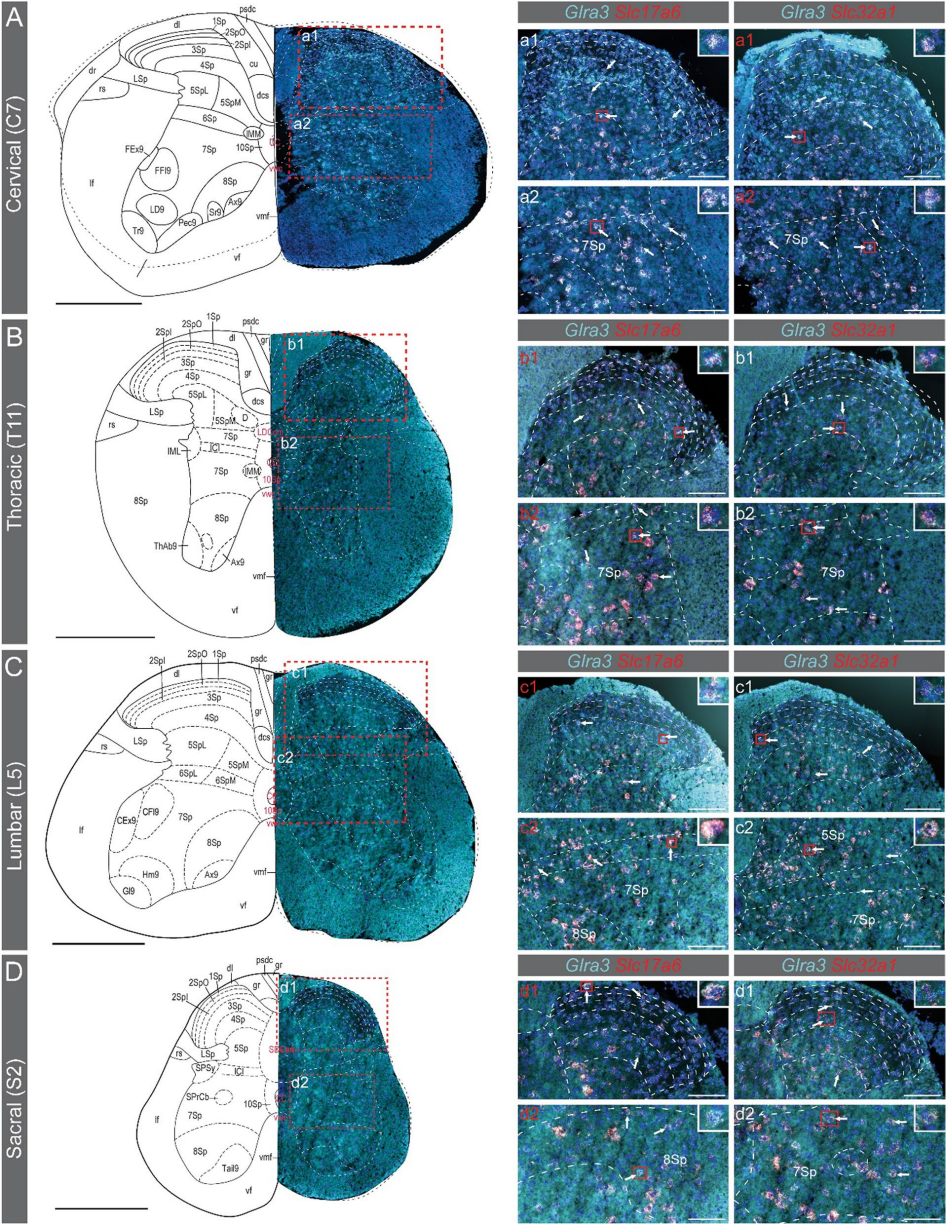
Spatial expression analysis of *Glra3* in the female spinal cord. The spatial *Glra3* mRNA expression was examined with RNAscope using probes for *Glra3 (teal), Slc17a6 (red)* and *Slc32a1 (red). Glra3* was expressed in the (**A**)cervical (C7), (**B**) thoracic (T11), (**C**) lumbar (L5) and (**D**) sacral (S2) divisions of the spinal cord (**A**–**D**). Overlap with *Slc17a6* and *Slc32a1*expressions could be observed in all divisions (a1, a2, b1, b2, c1, c2, d1, d2). Expression was found in the dorsal and ventral horns in all divisions. *Glra3* could be detected in the ventral horn in the cervical, lumbar and sacral divisions. Illustrations of the spinal cord divisions in **A**–**D** are modified from https://mouse.brain-map.org/experiment/siv?id=100050402&imageId=101006525&imageType=atlas. The red dashed squares in **A**–**D** indicate approximately the enlarged images in a1-d2 (labelled with white text). No overview images are shown for the enlarged images labeled with red text. **A**–**D** Scale bars: 500 µm, a1–d2: scale bars 100 µm. White arrows denote examples of co-expression. Grey line in a1-d2 indicates boarder for lamina II_outer_ and lamina II_inner_. 5Sp = lamina 5, 7Sp = lamina 7, 8Sp = lamina 8. For all other abbreviations, please see https://mouse.brain-map.org/experiment/siv?id=100050402&imageId=101006525&imageType=atlas

**Fig. 6:**
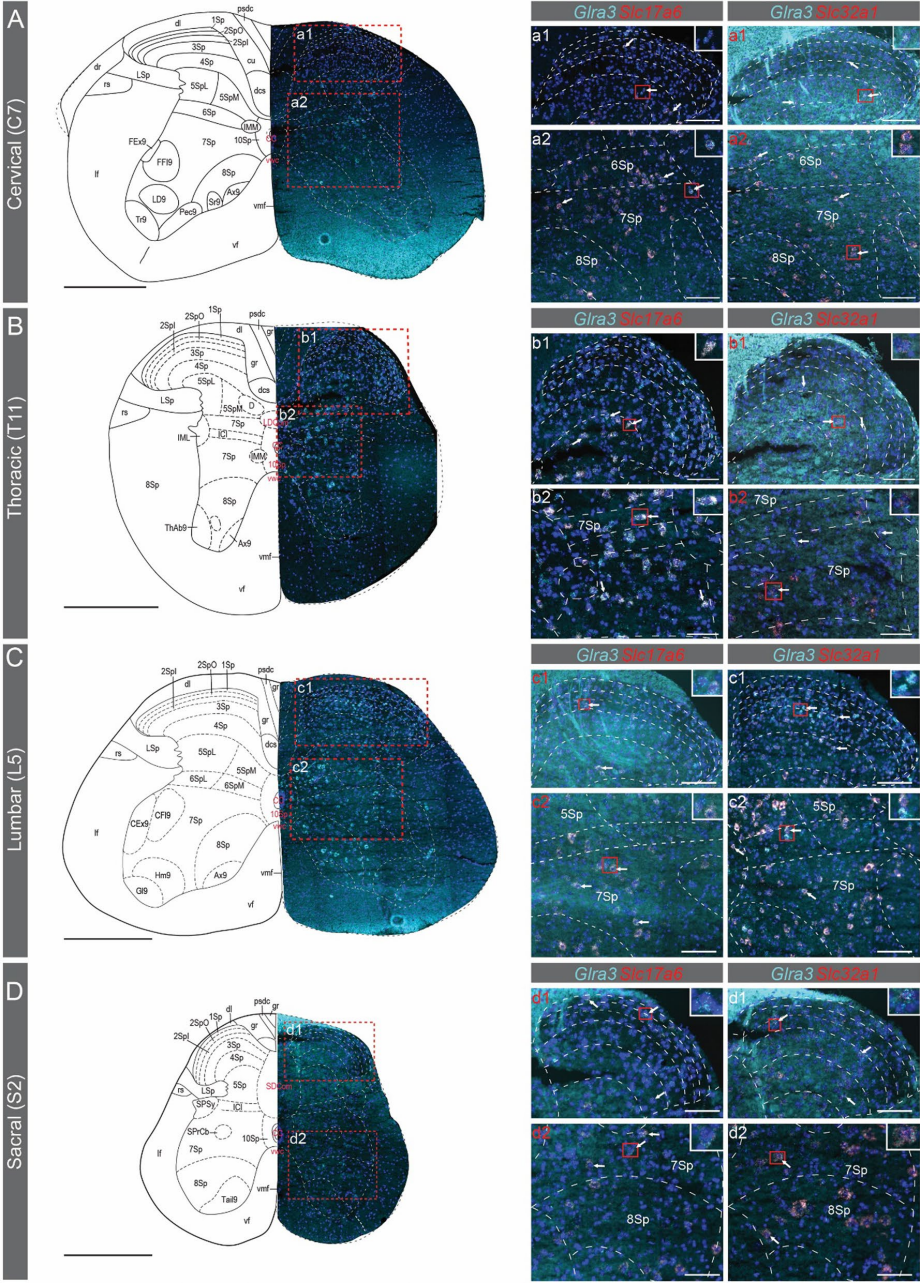
Spatial expression analysis of *Glra3* in the male spinal cord. The spatial *Glra3* mRNA expression was examined with RNAscope using probes for *Glra3 (teal), Slc17a6 (red)* and *Slc32a1 (red). Glra3* was expressed in the (**A**) cervical (C7), (**B**) thoracic (T11), (**C**) lumbar (L5) and (**D**) sacral (S2) divisions of the spinal cord (**A**–**D**). Overlap with *Slc17a6* and *Slc32a1* expressions could be observed in all divisions (a1, a2, b1, b2, c1, c2, d1, d2). *Glra3* expression was found in the dorsal and ventral horns in all divisions. *Glra3* could be detected in the ventral horn in the cervical, lumbar and sacral divisions. Illustrations of the spinal cord divisions in **A**–**D** are modified from https://mouse.brain-map.org/experiment/siv?id=100050402&imageId=101006525&imageType=atlas. The red dashed squares in **A**–**D** indicate approximately the area displayed in a1–d2 (labelled with white text). No overview images are shown for the enlarged images labeled with red text. **A**–**D** Scale bars: 500 µm, a1–d2: scale bars 100 µm. White arrows denote examples of co-expression. Grey line in a1-d2 indicates boarder for lamina II_outer_ and lamina II_inner_. 5Sp = lamina 5, 6Sp = lamina 6, 7Sp = lamina 7, 8Sp = lamina 8. For all other abbreviations, please see https://mouse.brain-map.org/experiment/siv?id=100050402&imageId=101006525&imageType=atlas

In the spinal cord of both females and males (Figs. 5, 6), *Glra3* was found in the dorsal horn of cervical (Figs. 5A, 6A), thoracic (Figs. 5B, 6B), lumbar (Figs. 5C, 6C) and sacral (Figs. 5D, 6D) divisions, with co-expressions of *Slc17a6* and *Slc32a1* detected in all divisions. Lastly, in the cervical, lumbar and sacral divisions, *Glra3* expression was detected in the ventral horn (Figs. 5C, D, 6C, D). In conclusion, spatial validations of *Glra3* expression verified that *Glra3* is expressed in all areas identified from the single-cell and bulk analyses. Co-localization of *Glra3* with *Slc17a6* was identified in all brain and spinal cord areas, except for the hippocampus, and with *Slc32a1* in all areas, except for the female thalamus.

## Discussion

Using three different mRNA-based methods, we here report that the glycinergic receptor unit *Glra3* is expressed in central nervous system areas such as the cortex, amygdala, striatum, hypothalamus, thalamus, hippocampus, brainstem, and spinal cord. Furthermore, we identified that male mice display higher levels of *Glra3* in the above listed areas, with the exception of the spinal cord. In all central nervous system areas, except for the hippocampus, *Glra3* expression overlapped with *Slc17a6* expression, whereas co-expression of *Glra3* and *Slc32a1* was found in all of the targeted areas except for the female thalamus.

### *Glra3* is expressed in several areas in the central nervous system

In the brain, GLRA3 exists as two isomers, namely the shorter GLRA3K and the longer GLRA3L (additional 8A exon), with the latter being the dominant variant in the mouse brain [25]. In our analyses, the *Glra3* qRT-PCR primers and the RNAscope probes targeted the nucleotide sequence outside the splicing area (primers: exon 9–10; probes: exon 1–8), meaning our analyses captured the expression of both isomers.

The Human Protein Atlas project has mapped human *GLRA3* [26] and mouse *Glra3* [27] (https://www.proteinatlas.org/ENSG00000145451-GLRA3/brain) to several areas in the central nervous system. In humans, *GLRA3* has been detected in the cerebral cortex, amygdala, hypothalamus, thalamus, hippocampal formation, midbrain, basal ganglia and the brainstem (pons and medulla oblongata) [26]. In mice, the Human Protein Atlas project could locate *Glra3* to the olfactory bulb, cerebral cortex, amygdala, hypothalamus, thalamus, hippocampal formation, midbrain, basal ganglia, brainstem (pons and medulla) and the cerebellum [27], similar to the expression pattern displayed in the Allen Mouse Brain Atlas (https://mouse.brain-map.org/experiment/show/73788474). These previous findings are coherent with our results (Table 2).

**Table 2:** Summary of *Glra3* expression in the central and peripheral nervous system

Areas	scRNA-seq^£^	qRT-PCR	RNAscope
Cortex	+	+	+
Amygdala	+	+	+
Striatum	+	+	+ (males)
Pallidum	+	NA	+
Hypothalamus	+	+	+
Pituitary gland	NA	+	NA
Hippocampus	+	+	+
Brainstem	+	+	+
Cerebellum	ND	+	ND
Spinal cord	+	+	+
Dorsal root ganglia	ND	NA	NA

+ expression, NA not analyzed, ND not detected. ^£^Zeisel et al. (2018) dataset [22]

Previous GLRA3 studies have mainly focused on what function the subunit has in the brain, i.e. in the cortex [19], striatum, nucleus accumbens [15, 19], hippocampus [19, 25, 28] and the brainstem [17, 29]. For instance, McCracken et al. (2017) showed that GLRA3-containing GlyRs are found in various areas of the forebrain. Additionally, when performing whole-cell recordings on *Glra3*^*−/−*^ mice, it was reported that these mice lacked tonic inhibition in the forebrain. These findings indicate that *Glra3* participates in tonic inhibition in the prefrontal cortex and in both the dorsal striatum and nucleus accumbens [19]. San Martin et al. (2021) investigated the potential role of the GLRA3 subunit in ethanol sensitivity by focusing on the nucleus accumbens [15]. They concluded that GLRA3 is expressed in low levels in the mouse nucleus accumbens [15]. Our qRT-PCR analysis detected *Glra3* in both the cortex and striatum, with higher levels found in male mice compared with female mice. Importantly, the dissected female striatum samples only contained caudate putamen, while the male striatum samples contained both caudate putamen and nucleus accumbens. This discrepancy could explain the difference in expression levels in male and female striatum. However, our RNAscope analysis identified a few *Glra3* positive cells in males but none in females, suggesting that *Glra3* expression may show a sex-dependent difference in striatum.

Earlier findings reported by Eichler et al. (2009) demonstrate that the expression of the GLRA3L splice variant is dominant in mice [25], but in temporal lobe epilepsy, the shorter splice variant (GLRA3K) was upregulated. Through these findings, Eichler et al. (2009) concluded that both splice variants are located on glutamatergic (3L) and GABAergic (3K) synaptic terminals [25]. Two of our transcriptional analyses, where the excitatory and inhibitory characteristics were examined, also disclosed that *Glra3* co-expresses with both an excitatory and an inhibitory marker. Schaefermeier and Heinze (2017) have also reported expression of murine *Glra3* in the hippocampus [28], in a similar expression pattern as was observed herein in female and male mice (Figs. 3 and 4).

All our transcriptional *Glra3* analyses mapped expression in the caudal brainstem (medulla and pons). This is consistent with previous immunostainings performed by Manzke et al. (2010), in which ubiquitous GLRA3 expression was detected in the brainstem [17]. The GLRA3 subunit has also been suggested to have a potential mechanism in mediating the presynaptic modulation of glycine release in the hypoglossal nucleus [29].

### *Glra3* is widely expressed in the spinal cord

Previous PCR expressional analysis of *Glra3* in the spinal cord reported that the gene is detected in the dorsal, but not in the ventral spinal horn [8]. In addition to the dense expression of *Glra3* seen in the dorsal horn, our RNAscope analysis revealed that *Glra3* was also detected in the ventral (with a majority medioventrally) horns of the cervical, lumbar and sacral divisions. Therefore, a broader expression pattern of the *Glra3* gene was displayed when compared to an earlier report [8]. In Groemer et al. (2022), the division of the spinal cord that was being analyzed was unspecified, suggesting that the ventral *Glra3* expression might have been missed. Expression of *Glra3* in the ventral horn has been shown by the Allen Institute, which is consistent with our findings. The spatial *Glra3* analyses also showed that *Glra3* overlaps with sub-populations expressing *Slc17a6* or *Slc32a1*. Using the Zeisel et al. (2018) and Häring et al. (2018) datasets [22, 30], we also found *Glra3* expression in both the excitatory SCGLU10 and Glut9, as well as the inhibitory Gaba8-9 clusters, further demonstrating the broad expression pattern of *Glra3*. Other studies have instead investigated GLRA3 expression in the spinal cord using immunostaining [12, 13], where its detection was restricted to the dorsal horn. The differences in the subunit’s protein expression, exhibited by immunostaining, compared to our RNAscope analysis may have been a result of not all *Glra3* units being translated into protein [31].

### *Glra3* expression in visceral organs

In this study, the *Glra3* expression in visceral organs was investigated with bulk qRT-PCR. Raw Ct-values (indicating expression) of *Glra3* could be detected in a few visceral organs, namely the heart, lung, spleen, kidney and testes. However, *Glra3* expression in these organs was difficult to detect using qRT-PCR compared with tissues collected from the central nervous system. The RFU was low, unstable amplification and melting curves were obtained, and many cycles (40–45 cycles) were needed to obtain a Ct-value, making it difficult to separate the proper amplification of *Glra3* in the visceral organs from background noise and non-specific amplification (e.g. primer-dimer). As a result, we cannot confidently conclude that *Glra3* is expressed in these visceral organs. Furthermore, chemical contamination, cycle-to-cycle variability and random noise are systematic errors that have been reported to affect results obtained with qRT-PCR [32]. These interferences could possibly explain the variability in detection levels in some of the tested tissues. However, our findings in mice are reasonably consistent with what the Human Protein Atlas project and Genotype-Tissue Expression project have reported on the *GLRA3* mRNA in humans [26]. The Human Protein Atlas project reports low levels of *GLRA3* mRNA in adrenal gland, pancreas, testes, female breast tissue, smooth muscle tissue, thymus, lymph nodes and tonsil tissue. Meanwhile the Genotype-Tissue Expression project reports *GLRA3* expression in the small intestine, testes and female breast tissue in 20–69 years old females and males using RNA sequencing (https://www.proteinatlas.org/ENSG00000145451-GLRA3/tissue), indicating inconsistencies in mRNA levels in the visceral organs. This inconsistency could be due to low levels of *Glra3,* the rate of mRNA turnover, or the point of transcription in which the tissues were harvested [33]. Therefore, what role *Glra3* has in visceral organs remains unknown.

### Methodological considerations

In Zeisel et al. [22] the specific regions included in what was labelled as cortex were not clearly specified, making it unclear if the entire cortex was included or merely substructures. Herein, in the bulk qRT-PCR, the cortex includes the main olfactory bulb, accessory olfactory bulb, anterior olfactory nucleus, orbital cortex and the frontal association cortex. Direct comparisons to the dataset of Zeisel et al. [22] are therefore limited. Moreover, in contrast to the males’ striatum samples, the dissected female striatum only contained caudate putamen and not nucleus accumbens, which could likely explain the differences in expression level in the striatum between females and males. Finally, amygdala, thalamus and pituitary gland were dissected from animals with the same housing and background, whereas the other qRT-PCR analyzed specimens were dissected from two different cohorts [34, 35]. However, the same expression pattern between females and males was revealed independent of this.

## Conclusions

We herein conclude that *Glra3* can be found in the cortex, amygdala, striatum, hypothalamus, hippocampus, brainstem and the spinal cord in female and male mice. The expression pattern was verified using three different mRNA-based methods. Furthermore, our analysis revealed that male mice display higher levels of *Glra3* in the cortex, amygdala, hypothalamus, thalamus, hippocampus, cerebellum and the brainstem than females. Based on the expression patterns, future analyses may investigate the functional role of GLRA3 in regulating somatosensory modalities, such as pruriception, and further address the role of the subunit in nociception, both in the brain and in the spinal cord.

## Methods

### Preprocessing of Zeisel et al. (2018) single-cell RNA sequencing dataset

The expression of *Glra3* in the nervous system was investigated in the Zeisel et al. (2018) scRNA-seq dataset [22]. The dataset ‘l5_all.loom’ was acquired from http://linnarssonlab.org/ and contains expression data of 27,998 genes in 160,796 single-cells from Vgat-Cre; *tdTomato* mice (with CD-1 and C57BL/6J background) to target inhibitory neurons and Wnt1-Cre; *R26Tomato* mice (with C57BL/6J background) to isolate neurons in the peripheral and enteric nervous systems. The scRNA-seq data was obtained using the 10X Genomics method. The dataset was analyzed using SCANPY 1.9.1 [36] in Python 3.8.8 in similarity as described before [37] and the full code can be found at https://github.com/HannahMWeman/glra3-expression-analysis-in-the-nervous-system. Firstly, all annotated neurons were isolated from the dataset to be used for basic preprocessing, resulting in 74,539 neurons and 27,998 genes. For gene filtering, all genes that were expressed in less than 3 cells (sc.pp.filter_genes) and all cells expressing less than 200 genes (sc.pp.filter_cells) were excluded, resulting in 74,529 neurons and 21,194 genes. Subsequently, for basic preprocessing, the metrics of the general gene expression and mitochondrial genes were calculated (SCANPY, pp.calculate_gc_metrics) [38]. By visualizing the distribution of the calculated metrics (SCANPY, pl.violin; Seaborn, jointplot), the cells with lower mitochondrial gene expression (SCANPY, ‘pct_counts_mt’ < 20), high total counts (SCANPY, ‘log1p_total_counts’ > 6.5), and distributed gene counts and broad gene capture (SCANPY, ‘logp_n_genes_by_counts’ > 6.0, ‘pct_counts_in_top_50_genes’ < 50) were isolated. The dataset did not contain External RNA Controls Consortium (ERCC) genes since the 10 × Genomics method does not include ERCC sequences, thus cells were not filtered based on expression criteria of these sequences. All the inclusion criteria resulted in 72,020 neurons and 21,194 genes to be used for the scRNA-seq analysis. Finally, the counts per cell was normalized to the medium number of counts (SCANPY, pp.normalize_per_cell) followed by normalization (SCANPY, pp.log1p).

### Single-cell RNA sequencing analysis of Glra3-expressing cells

The expressions of *Glra3*, as well as excitatory *Slc17a6* (*Vglut2*) and inhibitory *Slc32a1* (*Viaat*) markers, were visualized in the respective nervous system area (SCANPY, pl.DotPlot). Moreover, the prevalence of *Glra3* expression (*Glra3* considered expressed if log1p > 0.1) was calculated for the respective nervous system area. The expression patterns of *Slc17a6* and *Slc32a1* were more extensively examined in all of the *Glra3* neurons (a total of 628 neurons expressed *Glra3*) in the different nervous system areas by visualization (SCANPY, pl.DotPlot) and occurrence calculations.

### Animals

Protocols related to animal use in this study were approved by the local animal research ethical committee (Uppsala djurförsöksetiska nämnd) and followed the Swedish Animal Welfare Act [Svensk författningssamling (SFS) 2018:1192], The Swedish Animal Welfare Ordinance (SFS 2019:66) and the Regulations and General Advice for Laboratory Animals (SJVFS 2019:9, Saknr L 150). Both female and male C57BL/6J mice (Taconic, Denmark) were included in the analysis. The mice were housed with littermates in approximately 501 cm^2^ cages (maximum 5 mice in per cage), in room temperature ranging between 20 and 24 °C and humidity of 45–65% on a 12-h light:dark cycle with lights on at 6 am. All animals were provided food (Diet Pellets, Scanbur, Sweden) and tap water *ad libitum*. All procedures were planned and executed to minimize stress, and euthanasia was performed during the light period of the light:dark cycle.

### Tissue dissection for qRT-PCR

Tissues from five adult male C57BL/6J mice (10–14 weeks) had previously been collected and prepared as specified in [34, 35], where the striatum samples comprised of the caudate putamen and nucleus accumbens. The gDNA was previous collected and was a gift from Prof. Robert Fredriksson [39]. To add to this tissue mRNA panel, five adult female (14 weeks old) and five adult male (10–11 weeks old) C57BL/6J mice were euthanized via cervical dislocation, without prior treatment, during the light period. All tissues were collected on ice. The following tissues/areas were collected from the five females. The whole brain was scooped out leaving the majority of the main olfactory bulb in the scull. The pituitary gland was collected from sella turcica and the hypothalamus was collected from the brain using forceps. The brain was then placed in a mouse brain matrix (Activational Systems Inc., Warren, MI, USA; 1 mm) and sliced manually using matrices blades (ALTO Matrix Cutting Blades, AgnTho’s, Lidingö, Sweden). From coronal sections, the most frontal part of the cortex (herein cortex; containing main olfactory bulb, accessory olfactory bulb, anterior olfactory nucleus, orbital cortex, and frontal association cortex), caudate putamen (herein striatum), thalamus, hippocampus and amygdala were manually dissected with guidance from a mouse brain atlas [40] and collected. The whole cerebellum and brainstem were collected. Moreover, the following tissues were collected: spinal cord (late thoracic to sacral divisions), heart, small intestine, kidney, liver, lung, spleen, testes, thymus, and uterus. For the additional five male mice dissected for this paper, only amygdala, pituitary gland, and thalamus were dissected, as described for the females, to complement the other male panel [34, 35].

All tissues were collected within 10–15 min after sacrifice and stored in RNAprotect© Tissue Reagent (Qiagen, Germany) for 2 h at room temperature. All samples were then frozen at -80 °C before further processing.

### RNA extraction and cDNA synthesis

Total RNA was extracted using Absolutely RNA Mini kit (Qiagen, Germany) according to the manufacturer’s protocol. RNA concentrations were measured using ND-1000 spectrophotometer (NanoDrop Technologies, USA). The cDNA synthesis was performed using the Applied Biosystems High Capacity RNA-to-cDNA kit (Invitrogen, USA) following manufacturer’s instructions. 2 μg RNA template was used for the reaction and the cDNA samples were diluted to 10 ng/μl.

### Primer design and quantitative real-time PCR (qRT-PCR)

Primers were designed using Primer3 (*Glra3*) [41] or Beacon Design 8 (Premier Biosoft) (reference housekeeping genes). The primers were screened using BLAST and global alignments [42] to avoid primer pairs that can cause non-specific amplification. *Glra3* primers: forward 5′-*cggaagcttttgcactggag*-3′, reverse 5′-*tggaaccacaccatccttgg*-3′*.* Reference housekeeping genes: *ribosomal protein L19* (*Rpl19*) forward 5′-*aatcgccaatgccaactc*-3′, reverse 5′-*ggaatggacagtcacagg*-3′, *Peptidylprolyl isomeras A* (*Cyclo*) forward 5′-*tttgggaaggtgaaagaagg*-3′, reverse 5′-*acagaaggaatggtttgatgg*-3′, *glyceraldehyde-3-phosphate dehydrogenase* (*Gapdh*) forward 5′-*gccttccgtgttcctacc*-3′, reverse 5′-*gcctgcttcaccaccttc*-3′ and *actin-related protein 1B* (*Actb*) forward 5′-*ccttcttgggtatggaatcctgtg*-3′, reverse 5′-*cagcactgtgttggcatagagg*-3′.

*Glra3* expression was determined using qRT-PCR. Final volume for each reaction was 20 μl containing 3.6 μl 10 × DreamTaq Buffer (Thermo Fisher Scientific, USA), 0.2 μl of 20 mM dNTP mix (Invitrogen, USA), 1 μl DMSO, 0.5 μl SYBR Green (1:10,000, Invitrogen, USA) in 1 × TE buffer (pH 7.8), 0.08 μl DreamTaq polymerase (5 U/μl, Thermo Fisher Scientific, USA), 0.05 μl of forward and reverse primer (100 pmol/μl) and 5 μl cDNA (10 ng/μl). The volume was adjusted with sterile water. An iCycler real-time detection instrument (Bio-Rad, USA) was used with the following settings: initial denaturation for 30 s at 95 °C, 45 cycles of 10 s at 95 °C, 30 s at 55 °C for housekeeping genes or 55.7 °C for *Glra3* and 30 s at 72 °C. A melting curve was generated by heating from 55 to 95 °C with 0.5 °C increments at 10 s dwell time and a plate read at each temperature. All qRT-PCR were run in triplicates and a negative control and genomic DNA (10 ng/ul) were included on each plate. Cycle threshold (Ct) values were collected via the CFX Maestro (Bio-Rad, USA) and primer efficiencies were calculated via LinRegPCR software. The melting curves were compared with the negative control to verify that only one product was amplified. The delta Ct method for multiple reference genes (according to [23]) was used to calculate the normalized and relative mRNA expression of *Glra3,* and differences in primer efficiency were accounted for. Biological outliers in nervous system tissues (one for hippocampus and one for cerebellum from male mice) were removed using the Grubbs outlier test with α = 0.05 before proceeding and 45 cycles were set as cut-off. The same settings for the Grubbs outlier test and cycle threshold cut-off were used for the identification of biological outliers in the visceral organs. The following biological outliers were consequently removed; heart (two females and two males), lung (two males), liver (one female and one male), spleen (one female) and kidney (one female). The log2 fold difference to the genomic DNA expression of *Glra3* was calculated for all tissues and presented in the combined scatter-bar-plot graph (mean log2 difference against gDNA expression of *Glra3* ± SEM).

### In situ hybridization tissue preparation

Two adult female (13 weeks old) and two adult male (11–13 weeks old) C57BL/6J mice were intraperitoneally injected with 0.6 ml (1:1) Ketamin (Ketalar, 10 mg/ml, Pfizer, Sweden) and Medetomidine (Domitor, 1 mg/ml, Orion Pharma, Sweden) and subsequently perfused with autoclaved ice-cold 1 × PBS. To minimize the risk of contamination and altered gene expression, the following steps were performed as quickly as possible in autoclaved ice-cold 1 × PBS; the whole brains and all divisions of the spinal cord were dissected and cleaned from meninges, followed by embedding in optimal cutting temperature (OCT) medium (Bio-Optica, Italy) and snap-frozen on dry ice in -80 °C isopentane (Sigma-Aldrich, Germany). The tissues were stored in -80 °C until sectioning. The brains were cryo-sectioned (Leica Cryocut 1800, Leica, Germany) into 18 µm and the spinal cords into 14 µm sections and collected onto Superfrost Plus (Thermo Scientific, USA) slides. To prevent mRNA degradation and contamination, the completed series were stored at -21 °C until sectioning was completed. The slides were thereafter stored at -80 °C until the RNAscope Fluorescent Multiplex kit (Advanced Cell Diagnostics (ACD), USA, cat # 320850) protocol commenced.

### Fluorescent in situ hybridization

Fluorescent in situ hybridization was performed to target the expression of *Glra3* in various tissues using the RNAscope Fluorescent Multiplex kit (cat#: 320850, ACD, USA) in accordance with ACD guidelines for fresh frozen tissues with minor modifications [24] and as described previously [37]. In brief: the slides to be used were taken from -80 °C and immediately fixated in room temperature 4% PFA in 1 × PBS (Histolab, Sweden) for 15 min before being washed in autoclaved 1 × PBS for 2 min. The tissues were thereafter dehydrated in a step-wise increase of EtOH concentration; 3 min in 50%, 3 min in 70% and two times for 5 min in 100% (Merck KGaA, Damstadt, Germany). The slides were placed at room temperature for 5 min to dry whereafter a hydrophobic barrier was made around the slide area of interest (2 females and 2 males; brain: 2 sections/brain area of interest (Bregma 0.98, -1.34 and -6.84 mm [20] https://mouse.brain-map.org/experiment/thumbnails/100048576?image_type=atlas) from each animal; spinal cord: 4 sections/spinal cord division (cervical, thoracic, lumbar and sacral) from each animal [9] https://mouse.brain-map.org/experiment/siv?id=100050402&imageId=101006525&imageType=atlas) using an ImmeEdge pen (Vector Laboratories, USA). The sections were thereafter incubated in Protease IV for 40 min at room temperature, followed by washing three times for 5 min in autoclaved 1 × PBS. The treatment was followed by incubation with the target probes; *Glra3*: 490591-C2 and *Slc17a6 (Vglut2)*: 319171-C3 or *Slc32a1 (Viaat)*: 319191-C3 (1:50 in probe diluent, cat#: 300041) for 2 h at 40 °C in a hybridization oven (HybEZ™ II Oven, ACD, USA) (brain: 1 section/area from each animal per assay (Bregma 0.98, -1.34 and -6.84 mm [20] https://mouse.brain-map.org/experiment/thumbnails/100048576?image_type=atlas); spinal cord: 2 sections/division (cervical, thoracic, lumbar and sacral [9] https://mouse.brain-map.org/experiment/siv?id=100050402&imageId=101006525&imageType=atlas) from each animal per assay). The following amplification steps were performed at 40 °C in an oven and the sections were washed two times for 2 min in room temperature washing buffer between each amplification step; AMP 1-FL for 30 min, AMP 2-FL for 15 min, AMP 3-FL for 30 min and AMP 4-FL for 15 min. Lastly, the slides were washed two times for 2 min in washing buffer before 30 s incubation in DAPI and mounting in Anti-Fade Fluorescence Mounting Medium (Abcam, UK). The slides were covered with glass slides (Menzel-Gläser, Germany) and were left at 4 °C to dry. The slides were stored at this temperature until imaging.

### In situ image acquisition

Images of the RNAscope treated sections were acquired with wide field 20 × magnification using an Axio Imager.Z2 (Zeiss, Germany). Whole section images were acquired as tiles in the DAPI (150 ms), Cy3 (*Glra3* detection, 4000 ms) and Cy5 (*Slc17a6* or *Slc32a1* detection, 900 ms) channels. The images were handled for figure representation using the ZEISS ZEN 3.3 (blue edition) software, where the area outliners of the targeted brain structures were determined using the Allen mouse brain atlas (https://mouse.brain-map.org/experiment/thumbnails/100048576?image_type=atlas).

### Statistics

The obtained relative mRNA values for female and male mice were checked for normality using the Shapiro-Wilk test before proceeding with the appropriate analysis. Kruskal-Wallis test and Mann-Whitney U-test were used to determine expressional differences against the background and tissues/areas of the nervous system of both females and males (Additional file 1: Table S1). Furthermore, two-tailed Mann-Whitney U-test or unpaired t-test were used to calculate the difference between female and male mice for each tissue. Comparisons were considered significant at p < 0.05.

## Supplementary Information

**Additional file 1.** Additional figures, **Fig. S1-S2**. Additional Table, **Table S1**.

## Data Availability

The scRNA-seq data was acquired from Zeisel et al. (2018) scRNA-seq dataset [22], where the raw sequence data is deposited in the sequence read archive under accession SRP135960, available at https://www.ncbi.nlm.nih.gov/sra/SRP135960. The dataset ‘l5_all.loom’ was acquired from (http://linnarssonlab.org/). The dataset was analyzed using SCANPY 1.9.1 [36] in Python 3.8.8 in similarity as described before [37] and the full code can be found at https://github.com/HannahMWeman/glra3-expression-analysis-in-the-nervous-system. All qRT-PCR data generated or analyzed during this study are included in this published article and its additional files.

## Abbreviations

DRG: Dorsal root ganglia
Glra3: Glycine receptor alpha3 subunit
GlyR: Glycine receptor
GLYT2: Glycine transporter subtype 2
HPA: Hypothalamic–pituitary–adrenal
qRT-PCR: Quantitative real-time PCR
RFU: Relevant fluorescence unit
scRNA-seq: Single-cell RNA sequencing
Vglut2: Vesicular glutamate transporter 2 (*Slc17a6*)
Viaat: Vesicular inhibitory amino acid transporter (*Slc32a1*)
