# Supplementary material for: The glycine receptor alpha 3 subunit mRNA expression shows sex-dependent differences in the adult mouse brain
Source: BMC Neurosci. 2023 Jun 1;24:32. doi: 10.1186/s12868-023-00800-9 (PMC10233971; doi:10.1186/s12868-023-00800-9)
Supplement: Supplementary file 1 — Additional file 1. Additional figures, Fig. S1-S2. Additional Table, Table S1. [file 12868_2023_800_MOESM1_ESM.pdf]

Additional file information

The glycine receptor alpha 3 subunit mRNA expression shows sex-dependent differences in the adult mouse brain

Mikaela M. Ceder[1], Hannah M. Weman[1], Ebba Johansson[1], Katharina Henriksson[1], Kajsa A. Magnusson[1], Erika Roman[2,3] and Malin C. Lagerström[1*]

*Corresponding author

Email: Malin.Lagerstrom@igp.uu.se

This additional file contains Additional Fig. S1-2 and Additional Table S1, six pages.

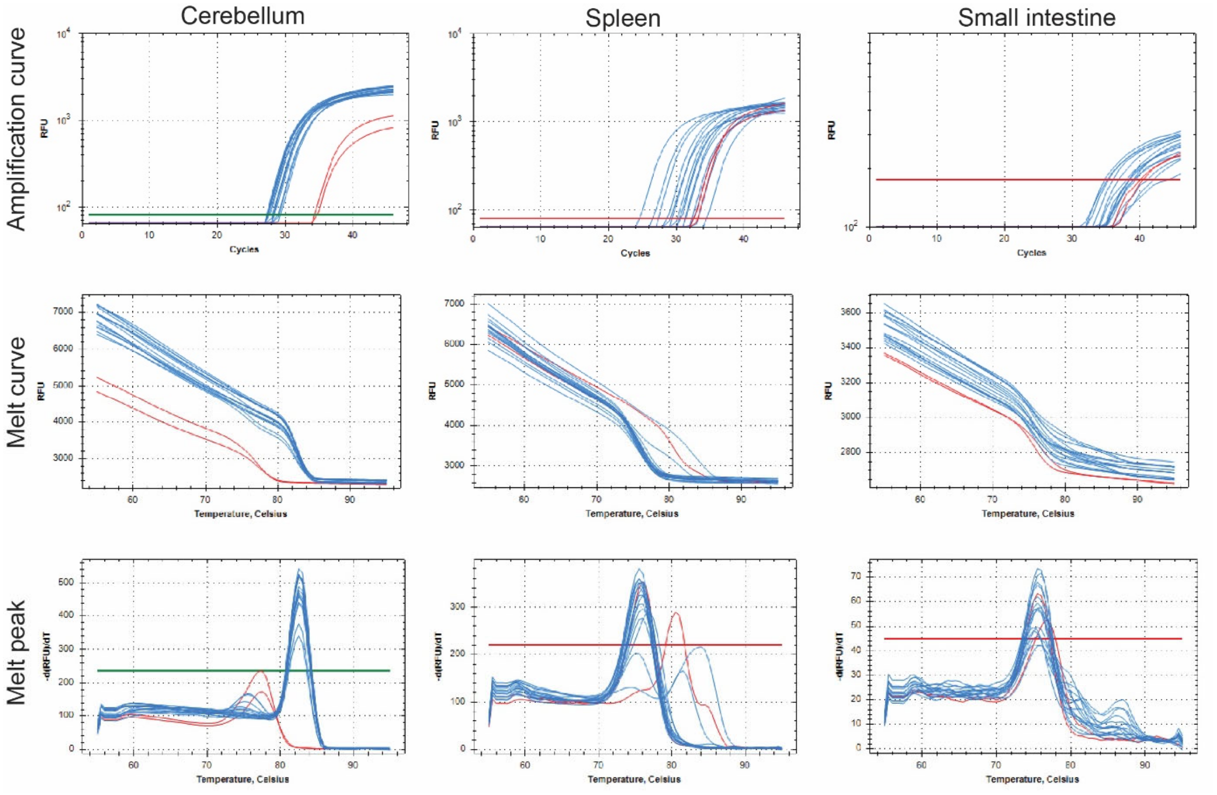

Fig. S1. **Representative images of logarithmic amplification curves, melt curves and melt peaks for cerebellum, spleen, and small intestine.**

Images of logarithmic amplification curves, melt curves, and melt peaks were collected from the CFX maestro software for cerebellum, spleen, and small intestine. There were difficulties in amplifying *Glra3* mRNA from tissues collected from visceral organs compared with tissues harvested from the central nervous system. Furthermore, several of the visceral organs were found not to express *Glra3,* or biological variances resulted in *Glra3* being detected in some mice. When examining the amplification curves, melt curves and melt peaks for the different tissues, it was clear that central nervous tissue was generally more cohesive. Each triplicate of the five replicates had good amplification of *Glra3* and the melt curve and peak showed one product with the correct length and little non-specific binding was amplified. This is illustrated in the left column in the figure showing a representative tissue (cerebellum) of five replicates (blue) and the negative control (red) obtained from female mice. Visceral tissues, on the other hand, often resulted in no specific amplification or non-cohesive replicates,

sometimes with great difference between the biological replicates. This is illustrated by the middle and right column of the figure showing the amplification curves, melt curves and melt peaks of *Glra3* in the spleen and intestine of male mice. The amplification curve of *Glra3* in spleen provided Ct-values that could be used for analyzing, but when examined, melt curves and melt peaks had the same melting temperature as the negative control, suggesting that *Glra3* had not been amplified. Ct = cycle threshold, dRFU = change in fluorescent level (RFU), dT = per unit change in temperature, RFU = Relative Fluorescent Unit.

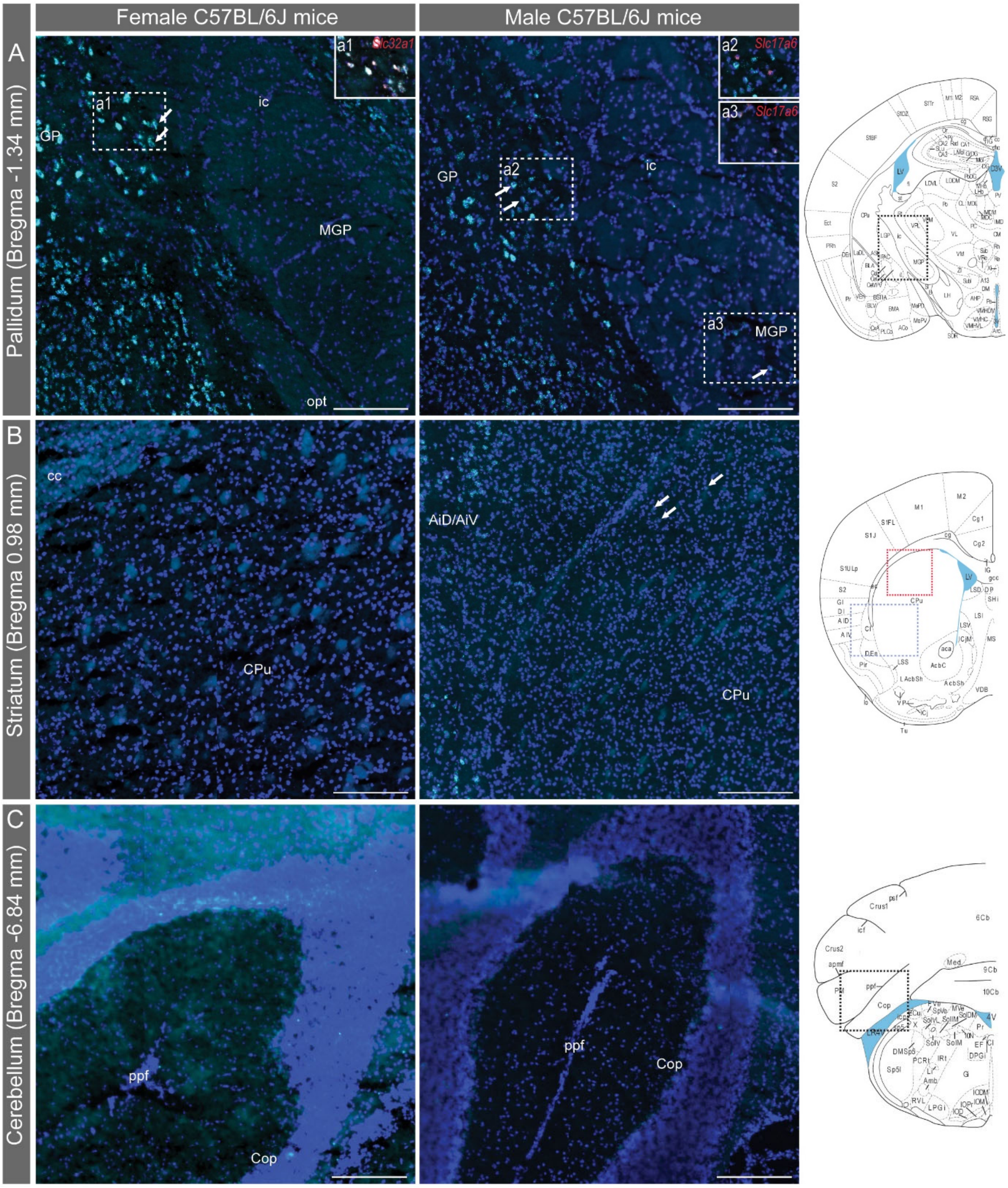

Fig. S2. Spatial analysis of the *Glra3* expression in pallidum, striatum and cerebellum. The spatial mRNA expression of *Glra3* was examined using RNAscope method (1) with probes for *Glra3 (teal), Slc17a6 (red),* and *Slc32a1 (red)*. (A) In pallidum (Bregma -1.34 mm), *Glra3* was detected in both sexes, and overlap (a1–a3) was observed with both *Slc17a6* and *Slc32a1* expressions. (B) In the striatum (Bregma 0.98 mm), no *Glra3* probes signal was

detected in females, while in males a few cells were found to express *Glra3.* (C) Meanwhile, in cerebellum (Bregma -6.84 mm), no *Glra3* probe signal could be detected regardless of sex. Illustrations to the right in A–C are adapted from https://mouse.brain-map.org/experiment/thumbnails/100048576?image_type=atlas (2). The black dashed squares indicate approximately the area displayed in A and C, while the red dashed square and the blue dashed square in B indicate approximately the area displayed for female and male pallidum, respectively. A–C: Scale bar 200 µm. White arrows denote examples of co-expression. AID = agranular insular cortex, dorsal, AIV = agranular insular cortex, ventral, cc = corpus callosum, Cop = copula of the pyramis, CPu = caudate putamen (striatum), GP = globus pallidus, ic = internal capsule, MGP = medial globus pallidus, opt = optic tract, ppf = prepyramidal fissure.

Table S1. Log2 fold difference of *Glra3* in different nervous system structures compared with background control. Kruskal-Wallis test (KW) was used followed by Mann-Whitney U-test (MWU), where appropriate, to determine differences between the areas of the nervous system against the background noise of the qRT-PCR runs. The comparisons were made separately for females (n = 5) and males (n = 5, outliers included).

Area	p-values (against background)			
	Females		Males	
	KW	MWU	KW	MWU
Cortex	< 0.0001	0.0295	< 0.0001	0.0016
Amygdala		0.0016		0.0016
Striatum		0.0295		0.0016
Hypothalamus		0.0016		0.0016
Thalamus		0.0016		0.0016
Pituitary gland		0.0109		0.0451
Hippocampus		0.0186		0.0016
Cerebellum		0.0295		0.0062
Brainstem		0.0062		0.0016
Spinal cord		0.0062		0.0109

References

1. Wang F, Flanagan J, Su N, Wang LC, Bui S, Nielson A, et al. RNAscope: a novel in situ RNA analysis platform for formalin-fixed, paraffin-embedded tissues. J Mol Diagn. 2012;14(1):22-9.

2. Lein ES, Hawrylycz MJ, Ao N, Ayres M, Bensinger A, Bernard A, et al. Genome-wide atlas of gene expression in the adult mouse brain. Nature. 2007;445(7124):168-76.
